# Repurposing of Medicines in the EU: Launch of a Pilot Framework

**DOI:** 10.3389/fmed.2021.817663

**Published:** 2022-01-10

**Authors:** Charlotte Asker-Hagelberg, Tomas Boran, Christelle Bouygues, Sini Marika Eskola, Laszlo Helmle, César Hernández, François Houýez, Helen Lee, Dimitra D. Lingri, Laurent Louette, Lydie Meheus, Wim Penninckx, Beata Stepniewska

**Affiliations:** ^1^Swedish Medical Products Agency, Uppsala, Sweden; ^2^Director of the Marketing Authorisation Section, State Institute for Drug Control (SUKL), Prague, Czechia; ^3^Regulatory Affairs Office, Human Medicines Division, European Medicines Agency (EMA), Amsterdam, Netherlands; ^4^European Federation of Pharmaceutical Industries and Associations (EFPIA), Brussels, Belgium; ^5^European Commission Directorate-General Research and Innovation, Brussels, Belgium; ^6^Alternate Representative of Spain in EMA Management Board, Spanish Agency of Medicines and Medical Devices (AEMPS), Madrid, Spain; ^7^Treatment Information and Access, European Organisation for Rare Diseases (Eurordis), Paris, France; ^8^European Commission Directorate-General for Health and Food Safety, Brussels, Belgium; ^9^Association Internationale de la Mutualité Pharmaceutical Working Group, Brussels, Belgium; ^10^European Confederation of Pharmaceutical Entrepreneurs, Brussels, Belgium; ^11^Anticancer Fund, Appointed Alternate of the Committee for Advanced Therapies of the European Medicines Agency (CAT/EMA) to Represent Patients, Strombeek-Bever, Belgium; ^12^Federal Agency of Medicines and Health Products (FAMHP), Brussels, Belgium; ^13^Medicines for Europe, Brussels, Belgium

**Keywords:** repurposing, off patent authorised medicines, COVID-19, patient access, European Union, repurposing observatory group, pilot launch

## Abstract

Repurposing of authorised medicines has been under discussion for a long time. Drug repurposing is the process of identifying a new use for an existing medicine in an indication outside the scope of the original approved indication. Indeed, the COVID-19 health crisis has brought the concept to the frontline by proving the usefulness of this practise in favour of patients for an early access to treatment. Under the umbrella of the Pharmaceutical Committee and as a result of the discussions at the European Commission Expert Group on Safe and Timely Access to Medicines for Patients (STAMP) a virtual Repurposing Observatory Group (RepOG) was set up in 2019 to define and test the practical aspects of a pilot project thought to provide support to “not-for-profit” stakeholders generating or gathering data for a new therapeutic use for an authorised medicine. The group's initial plan was impacted by the outbreak of the SARS-CoV-2 pandemic and the launch of the pilot needed to be postponed. This article describes the progress and the activities conducted by the group during this past and yet extraordinary 2020–2021 to keep the project alive and explores on the background of this topic together with the obvious opportunities this health crisis has brought up in terms of repurposing of medicines.

## Introduction

Medicine repurposing is the process of identifying and substantiating a new use for an existing medicine/active substance outside the scope of the original indications (1–3) as well as the process of allowing a medicinal product to broaden its position in a relevant market (excluding the extension of an authorised indication to those of a new age group or to another genetic mutation). It includes new therapeutic uses for existing medicines, different formulations of the same medicine, and/or creating new combinations of medicines or medicines with medical devices. Repurposing of medicines is part of the routine research portfolio of both the pharmaceutical industry and academic institutions in the search for solutions for those conditions with unmet medical needs (4, 5) including aspects related to sustainability and patient access.

The perspective for repurposing is quite different when one considers finding a new use for (i) an active substance that has never been authorised (ii) a medicine that is still within intellectual property^1^ or regulatory data protection (6) or (iii) a well-known medicine that is out of any protection period. While pharmaceutical companies may find a commercial interest in pursuing a non-clinical and clinical development in the first two cases, they are less likely to carry out such development for out-of-protection medicines (7–10). In the third case, the current environment (regulatory and market access) does not encourage pharmaceutical companies to further explore existing opportunities in repurposing. A direct consequence is that pharmaceutical companies usually explore repurposing medicines that are still within the period of regulatory data protection. Other parties, including academic institutions and learned societies, are more willing in general to explore repurposing options when medicines are out of these protection periods (11). However, this academic research rarely has an impact in terms of regulatory recognition of a new use and indication. Academic sponsors usually do not intend to become marketing authorisation holders, and may have limited knowledge about regulatory requirements. In support, the EU Commission has initiated a regulatory science curriculum project called STARS^2^ (Strengthening Training of Academia in Regulatory Science) (12, 13). In addition, the current regulatory pathways do not foresee submission of data by parties that are not intending to be a marketing authorisation holder. This can mean that medicines are used outside their authorised uses (off-label) and official clinical guidelines might recommend their use based on available evidence, despite not being formally authorised (14, 15).

## Regulatory Context and Proposed Way Forward

There is a need to find a way for new and promising indications that will benefit patients in all EU member states in fulfilling an unmet medical need to be included on-label. Not converting off-label use into on-label use has a number of negative consequences. First of all, this means that this use is not included in the regulatory documents (i.e., summary of product characteristics and patient information leaflet). Hence, patients are not informed about the appropriate conditions of the particular use and warnings in the patient information leaflet. Patient access to potentially effective treatments may also be hampered in the absence of a formal authorisation. Moreover, when patients cannot lean on written and assessed information about the use it may lead to distrust or a sense of insecurity in the treatment. It may also have reimbursement impacts in some countries, but not in others (16). Secondly, established medicines that are no longer standard of care may also be withdrawn from the market without the full awareness of the potential off-label uses and patient needs, as these are not officially included in the label. Finally, repurposing of well-known, established medicines into new, sometimes higher priced medicines invariably introduces tension in the health systems that play against access. In other cases of investing in repurposing and adding a new indication to medicines' label, this effort is not recognised by market access mechanisms and prices remains the same as for other similar products without repurposed indication. It has therefore been recommended to find solutions to facilitate bringing new uses for medicines “on-label” by developing a collaborative framework between not-for-profit and academic organisations, patients organisations, pharmaceutical industry, health technology assessment bodies, payers, and regulators (17).

Within this context, a multistakeholder subgroup of the European Commission's Expert Group on Safe and Timely Access to Medicines for Patients (STAMP) started discussing a proposal to develop a framework for the repurposing of established medicines in the European Union. This group was made up of representatives of the European Commission, the European Medicines Agency (EMA), and National Competent Authorities (NCA) of several Member States as well as representatives of patients associations, research organisations and pharmaceutical industry associations and payers. On 11 July 2019, the Pharmaceutical Committee endorsed the proposal (18) of a framework to provide visible support to “not-for-profit” stakeholders, termed “champions,” who are generating or gathering data in accordance with regulatory standards for a new therapeutic use for an authorised active substance or medicine. The framework is only intended for medicines already out of intellectual property, data or marketing protection. The “champion” would typically be a “not-for-profit” organisation, for example an entity or a person from a charity or patient group, academic unit, learned society, research funder or payer. The framework builds on existing regulatory tools, namely support from the EU-Innovation Network (EU-IN) and scientific and/or regulatory advice provided through EMA and/or a NCA to provide guidance to the champion. The framework foresees that the champion would in time liaise with the marketing authorisation holder (MAH) of the potentially repurposed medicine, with the expectation that the latter can initiate an application for the new indication of use (i.e., on-label use) of the medicine by applying for variation, line extension or new marketing authorisation to a regulatory authority.

**Figure 1:**
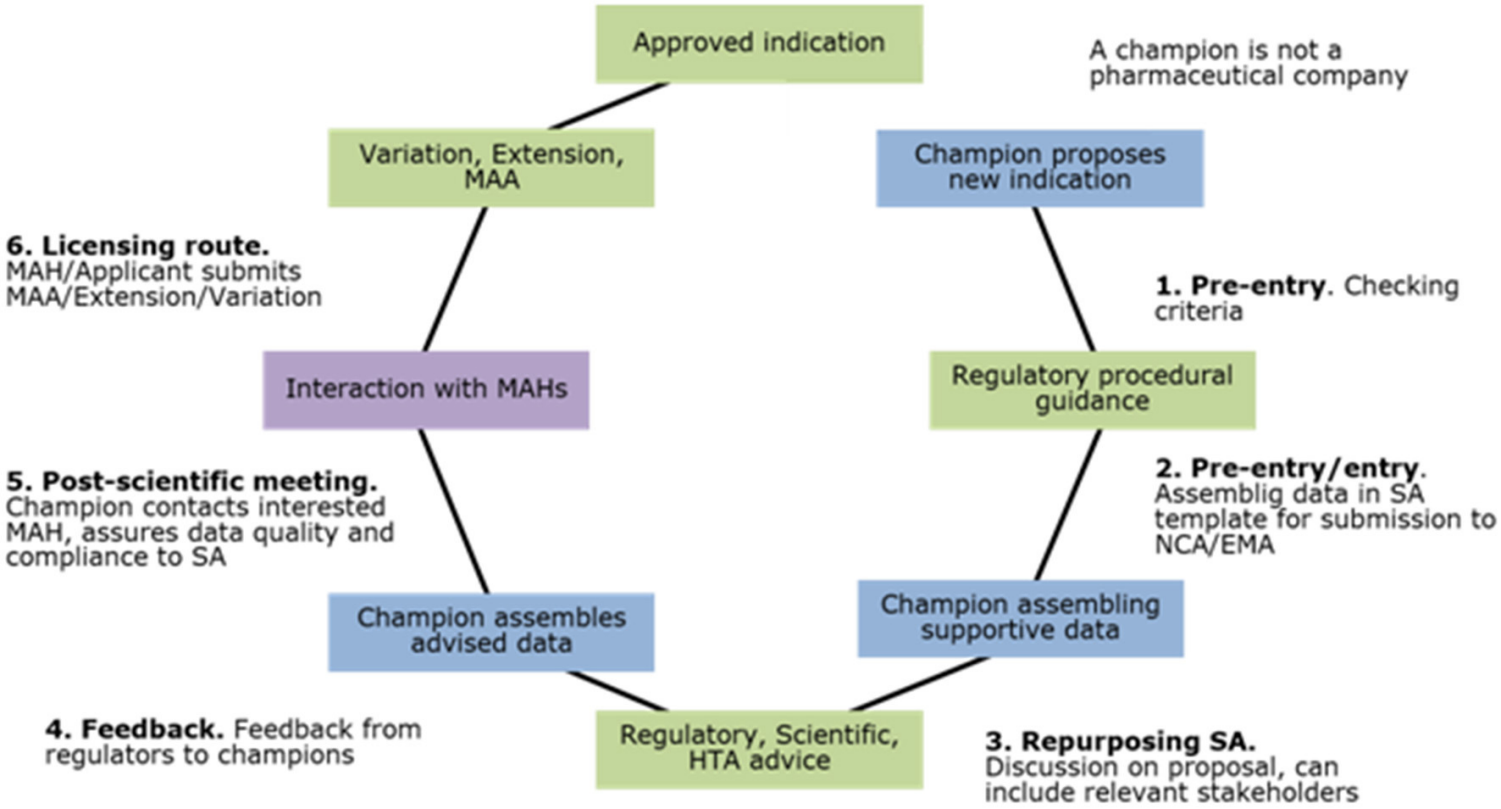
Regulatory framework for the repurposing of medicinal products out of data and patent protection by not-for-profit-organisations.

The next step is to establish a pilot to test the core components of the framework. This exercise would explore, among other aspects, the feasibility of producing and/or gathering by the “champions” of the required information for the regulatory approval, the adequacy of the current regulatory pathways as well as the suitability of current regulatory tools for the repurposing, and/or the challenges of the current systems for providing incentives to champions and MAHs to participate in the repurposing framework. Figure 1 gives an overview of the process of the pilot project.

For these purposes, a Repurposing Observatory Group (RepOG)^3^ was established in July 2019 with the aim of defining the practical aspects of the implementation of the pilot phase and to report on the success and opportunities of the project. The RepOG initiated its activities by further developing the framework: (i) establishing contact points and the steps for involvement of EMA's scientific advice working party (SAWP) and the EU-IN, (ii) developing materials such as a template for “champions” seeking scientific advice, (iii) a Question & Answer document, and (iv) a dissemination plan targeting potential “champions” as well as MAH with medicines that may be, at some point, be subject to the repurposing framework.

## Discussion

March 2020 was the planned date for launching the pilot in the EU. The commitment of the regulatory system was demonstrated by: the involvement of several NCAs and EMA via the EU-IN and the inclusion of the repurposing pilot in the EU-IN annual workplan; the inclusion of proposed actions to support repurposing in the EMA regulatory science strategy; and EMA's recent announcement that protocol assistance for academic organisations developing orphan medicines will now be free of charge (19). It can also be noted that EMA adapted the policy on conflicts of interest for patients/experts who would engage in drug repurposing or whose organisation would and which entered into force on 1 January 2021.

Unfortunately, we all know now that March 2020 is not going to be remembered because of the launch of a pilot project for repurposing. March 2020 will be kept in our minds because the SARS-CoV-2 virus causing a disease named COVID-19 struck our health systems, our societies and our citizens unlike anything else in the last 65 or more years. The EU and global medicines agencies had to reorganise themselves to tackle the health crisis and some ongoing activities were temporarily suspended or delayed. Among them, the launch of the repurposing pilot, the finalisation of materials to raise awareness of the repurposing project, and the regular meetings with stakeholders were postponed.

While the initial launch plans were interrupted by the COVID-19 pandemic, to some extent the pilot has started by itself. The health crisis has highlighted the massive opportunities for repurposing, acting as a kind of accelerator. This has become evident as knowledge and hypotheses about COVID-19 have emerged rapidly. EMA and the NCAs have been approached by sponsors in health care for central and national scientific advice on clinical trials in possible repurposing projects. In August 2020 there were more than 300 substances and 1,000 clinical trials recorded in a database produced by the Anticancer Fund^4^ (20). Moreover, in February 2021 there were around 1,600 drugs by over 1,000 sponsors in clinical trials recorded in a database by Informa Pharma Intelligence^5^ It is clear that repurposing of well-known medicines for COVID-19 disease is done extensively. Outside the scope of COVID-19, repurposing also seems to have taken off (21–23).

First, it has shown the various angles of repurposing, from medicines that have never been approved before but have been the subject of clinical development in other indications (like remdesivir), medicines approved but still within protection periods (e.g., tocilizumab, sarilumab or baricitinib, among others) to medicines out of protection periods (e.g., hydroxychloroquine, lopinavir, interferon or dexamethasone, among others).

Secondly, it has shown the different perspectives from where clinical proof or evidence may come. In fact, before the COVID-19 crisis, roughly 80% of the clinical trials approved were sponsored by pharmaceutical companies and only 20% by academic sponsors. During this crisis, these numbers were more or less reversed. Furthermore, the collaboration between public and private partners has also been remarkable, with pharmaceutical companies donating their medicines to ensure the fastest possible generation of evidence. Without doubt, saturation of health systems did not create the easiest environment for clinical research and clinical trials had to be designed and be adapted pragmatically to that situation. This has prompted a debate on the suitability of such clinical trials to offer meaningful pieces of evidence. However, the contribution of experiences in clinical trials such as the RECOVERY (24), comparing the use of dexamethasone or the usual care in hospitalised COVID-19 patients, SOLIDARITY (25), comparing the use of four repurposed antiviral drugs (remdesivir, hydroxychloroquine, lopinavir or interferon beta 1a) and the standard of care in hospitalised COVID-19 patients- and REMAP-CAP^6^ platform trial comparing multiple treatments for community-acquired pneumonia—should be highlighted and constitute an incredibly useful piece of evidence that complement others. These three examples of clinical trials are platform trials which compare head-to-head multiple treatments and where a “champion” acts as the sponsor.

Finally, it is also important to note that some of these results have prompted a timely recognition of a repurposed use. In July 2020, EMA initiated—at the request of the EMA Executive Director—a review (26) by the Committee for Medicinal Products for Human Use (CHMP) of the dexamethasone results from the RECOVERY trial. The CHMP issued an opinion recommending conditions for the safe and effective use of dexamethasone in adults and adolescents (from 12 years of age and weighing at least 40 kg) who require supplemental oxygen therapy. What is important is that, in view of the emergency situation, the CHMP exceptionally defined in its recommendation the conditions of use of dexamethasone in COVID-19 patients. Companies marketing dexamethasone medicines that wished to request this new use to be added to their product's licence could then base their request on the CHMP recommendation when submitting an application to national medicines agencies or to EMA (27). Whilst this mechanism was used in the context of an emergency situation, the importance of a scientific dialogue between partners and authorities for repurposing projects is pivotal. This is intended to be developed in the context of dedicated Scientific Advices as part of the pilot project.

## Conclusion

The subsequent waves of the COVID-19 pandemic have once again delayed the launch of this project, however, the RepOG has resumed its activities and a proposal for a new date was agreed, the pilot project has finally been launched on the 28^th^ of October (28). In addition to COVID-19 acting as an accelerator, repurposing is also described in the Pharmaceutical Strategy for Europe (29). Moreover, the proposal is also complementary with other important ongoing initiatives like the EU-IN workplan or the STARS project. Finding a smooth way for repurposing provides many opportunities for patients, health care professionals, cooperative and payers groups, and research institutions as well as for MAHs working in the innovation or generic/biosimilar side.

We recommend everyone to stay tuned.

## Data Availability Statement

Publicly available datasets were analysed in this study. This data can be found here: https://ec.europa.eu/health/documents/pharmaceutical-committee/stamp_en~https://www.ema.europa.eu/en/news/repurposing-authorised-medicines-pilot-support-not-profit-organisations-academia.

## Author Contributions

All authors listed have made a substantial, direct, and intellectual contribution to the work and approved it for publication.

## Author Disclaimer

The views expressed in this article are the personal views of the author(s) and may not be understood or quoted as being made on behalf of or reflecting the position of the regulatory agency/agencies or organisations with which the author(s) is/are employed/affiliated.

## Conflict of Interest

The authors declare that the research was conducted in the absence of any commercial or financial relationships that could be construed as a potential conflict of interest.

## Publisher's Note

All claims expressed in this article are solely those of the authors and do not necessarily represent those of their affiliated organizations, or those of the publisher, the editors and the reviewers. Any product that may be evaluated in this article, or claim that may be made by its manufacturer, is not guaranteed or endorsed by the publisher.

## Footnotes

^1^As per the World Intellectual Property Organisation (WIPO): Intellectual property (IP) refers to creations of the mind –everything from works of art to inventions, computer programs to trademarks and other commercial signs.

^2^ https://www.csa-stars.eu/

^3^RepOG composition at the time of the paper elaboration: Anticancer Fund (ACF), Association Internationale de la Mutualité (AIM), Federal Agency of Medicines and Health Products, Belgium (FAMHP), European Organisation for Rare Diseases (EURORDIS), European Federation of Pharmaceutical Industries and Associations (EFPIA), European Medicines Agency (EMA), European Commission (EC), European Confederation of Pharmaceutical Entrepreneurs (EUCOPE), Medicines for Europe, Spanish Agency of Medicines and Medical Devices (AEMPS), State Institute For Drug Control (SUKL), Swedish Medical Products Agency (MPA).

^4^Covid19db | ReDO Project (redo-project.org).

^5^ https://dataanalytics.citeline.com/coronavirus/

^6^ https://www.remapcap.org-adaptative

## References

[1] Ashburn TT Thor KB. Drug repositioning: identifying and developing new uses for existing drugs. Nat Rev Drug Discov. (2004) 3:673–83. 10.1038/nrd1468 15286734

[2] Langedijk J Mantel-Teeuwisse AK Slijkerman DS Schutjens MH. Drug repositioning and repurposing: terminology and definitions in literature. Drug Discov Today. (2015) 20:1027–34. 10.1016/j.drudis.2015.05.001 25975957

[3] Pushpakom S Iorio F Eyers PA Escott KJ Hopper S Wells A. Drug repurposing: progress, challenges and recommendations. Nat Rev Drug Discov. (2019) 18:41–58. 10.1038/nrd.2018.168 30310233

[4] Cha Y Erez T Reynolds IJ Kumar D Ross J Koytiger G. Drug repurposing from the perspective of pharmaceutical companies. Br J Pharmacol. (2018) 175:168–80. 10.1111/bph.13798 28369768 PMC5758385

[5] Frail DE Brady M Escott KJ Holt A Sanganee HJ Pangalos MN. Pioneering government-sponsored drug repositioning collaborations: progress and learning. Nat Rev Drug Discov. (2015) 14:833–41. 10.1038/nrd4707 26585533

[6] Article 14(11) of Regulation (EC) No 726/2004 OF THE EUROPEAN PARLIAMENT AND 242 OF THE COUNCIL of 31 March 2004 Laying Down Community Procedures for the Authorisation and Supervision of Medicinal Products for Human and Veterinary Use and Establishing a European Medicines Agency. Official Journal of the European Union. Available online at: https://eur-lex.europa.eu/legal-content/EN/TXT/PDF/?uri=CELEX:32004R0726&from=EN (accessed Dec 10, 2021).

[7] Breckenridge A Jacob R. Overcoming the legal and regulatory barriers to drug repurposing. Nat Rev Drug Discov. (2019) 18:1–2. 10.1038/nrd.2018.92 29880920

[8] Hernandez JJ Pryszlak M Smith L Yanchus C Kurji N Shahani VM. Giving drugs a second chance: overcoming regulatory and financial hurdles in repurposing approved drugs as cancer therapeutics. Front Oncol. (2017) 7:273. 10.3389/fonc.2017.00273 29184849 PMC5694537

[9] Langedijk J Whitehead CJ Slijkerman DS Leufkens HG Schutjens MH Mantel-Teeuwisse AK. Extensions of indication throughout the drug product lifecycle: a quantitative analysis. Drug Discov Today. (2016) 21:348–55. 10.1016/j.drudis.2015.11.009 26657087

[10] Sahragardjoonegani B Beall RF Kesselheim AS Hollis A. Repurposing existing drugs for new uses: a cohort study of the frequency of FDA-granted new indication exclusivities since 1997. J Pharm Policy Pract. (2021) 14:3. 10.1186/s40545-020-00282-8 33397471 PMC7780607

[11] Davies EH Fulton E Brook D Hughes DA. Affordable orphan drugs: a role for not-for-profit organizations. Br J Clin Pharmacol. (2017) 83:1595–1601. 10.1111/bcp.13240 28109021 PMC5465340

[12] Starokozhko V Kallio M Kumlin Howell Å Mäkinen Salmi A Andrew-Nielsen G Goldammer M. Strengthening regulatory science in academia: STARS, an EU initiative to bridge the translational gap. Drug Discov Today. (2020) 26:283–8. 10.1016/j.drudis.2020.10.017 33127567

[13] Gonzalez-Quevedo R Ziogas C Silva I Vegter R Humphreys A. Advancing development of medicines by academia and non-profit research organizations in the European Union. Nat Rev Drug Discov. (2020) 20:245–6. 10.1038/d41573-020-00205-x 33230307

[14] Bugani G Ponticelli F Giannini F Gallo F Gaudenzi E Laricchia A. Practical guide to prevention of contrast-induced acute kidney injury after percutaneous coronary intervention. Catheter Cardiovasc Interv. (2021) 97:443–50. 10.1002/ccd.28740 31967390

[15] Telmesani Moss A Chetrit M. Colchicine in Acute Pericarditis. American College of Cardiology. Available online at: https://www.acc.org/latest-in-cardiology/articles/2019/12/04/08/22/the-use-of-colchicine-in-pericardial-diseases (accessed October 05, 2019).

[16] Study on Off-Label Use of Medicinal Products in the European Union. (2017). Available online at: https://ec.europa.eu/health/sites/default/files/files/documents/2017_02_28_final_study_report_on_off-label_use_.pdf (accessed Dec 10, 2021).

[17] Verbaanderd C Rooman I Meheus L Huys I. On-label or off-label? Overcoming regulatory and financial barriers to bring repurposed medicines to cancer patients. Front Pharmacol. (2020) 10:1664. 10.3389/fphar.2019.01664 32076405 PMC7006723

[18] Proposal for a Framework to Support Not-For-Profit Organizations and Academia (institutions and individuals) in Drug Repurposing. Prepared by a Working Group of the Safe and Timely Access to Medicines for Patients (STAMP) Expert Group. (2019). Available online at: https://ec.europa.eu/health/sites/health/files/files/committee/pharm773_repurposing_annex_en.pdf (accessed Dec 10, 2021).

[19] Academia Developing Medicines for Rare Diseases to Receive Free EMA Scientific Advice. Available online at: https://www.ema.europa.eu/en/news/academia-developing-medicines-rare-diseases-receive-free-ema-scientific-advice (accessed June 23, 2020.

[20] Pantziarka P Vandeborne L Meheus L Bouche G. Covid19db – An online database of trials of medicinal products to prevent or treat COVID-19, with a specific focus on drug repurposing. (2020). [Preprint]. 10.1101/2020.05.27.20114371

[21] Polamreddy P Gattu N. The drug repurposing landscape from 2012 to 2017: evolution, challenges, and possible solutions. Drug Discov Today. (2019) 24:789–95. 10.1016/j.drudis.2018.11.022 30513339

[22] Parvathaneni V Kulkarni NS Muth A Gupta V. Drug repurposing: a promising tool to accelerate the drug discovery process. Drug Discov Today. (2019) 24:2076–85. 10.1016/j.drudis.2019.06.014 31238113 PMC11920972

[23] Pantziarka P Pirmohamed M Mirza N. New uses for old drugs. BMJ. (2018) 361:k2701. 10.1136/bmj.k2701 29945952

[24] Recovery Collaborative Group Horby P Lim WS Emberson JR Mafham M Bell JL. Dexamethasone in hospitalized patients with Covid-19. N Engl J Med. (2021) 384:693–704. 10.1056/NEJMoa2021436 32678530 PMC7383595

[25] Who Solidarity Trial Consortium Pan H Peto R Henao-Restrepo AM Preziosi MP Sathiyamoorthy V. Repurposed antiviral drugs for Covid-19 - interim WHO solidarity trial results. N Engl J Med. (2021) 384:497–511. 10.1056/NEJMoa2023184 33264556 PMC7727327

[26] EMA Starts Review of Dexamethasone for Treating Adults With COVID-19 Requiring Respiratory Support. Available online at: https://www.ema.europa.eu/en/news/ema-starts-review-dexamethasone-treating-adults-covid-19-requiring-respiratory-support (accessed July 24, 2020).

[27] Outcome of Art 5(3) Procedure - Product Information. Available online at: https://www.ema.europa.eu/en/documents/other/dexamethasone-covid19-article-53-procedure-proposals-product-information_en.pdf (accessed Dec 10, 2021).

[28] Repurposing of Authorised Medicines: Pilot to Support Not-For-Profit Organisations and Academia. Available online at: https://www.ema.europa.eu/en/news/repurposing-authorised-medicines-pilot-support-not-profit-organisations-academia (accessed October 28, 2021).

[29] Communication from the Commission to the European Parliament the Council the the European Economic Social Committee the Committee of the Regions. Pharmaceutical Strategy for Europe. (2020). Available online at: https://eur-lex.europa.eu/legal-content/EN/TXT/PDF/?uri=CELEX:52020DC0761&from=EN (accessed November 25, 2020).

